# Formulation of fortified instant weaning food from *Musa paradisiaca* (banana) and *Eleusine coracana*

**DOI:** 10.3389/fnut.2023.1203955

**Published:** 2023-06-15

**Authors:** Safreena Kabeer, Nagamaniammai Govindarajan, Preetha Radhakrishnan, Hanan F. Alharbi, Musthafa Mohamed Essa, M. Walid Qoronfleh

**Affiliations:** ^1^Department of Food Process Engineering, SRM Institute of Science and Technology, Kattankalathur, Chennai, Tamil Nadu, India; ^2^Department of Food Technology, Faculty of Engineering, Karpagam Academy of Higher Education, Coimbatore, India; ^3^Department of Food Process Engineering, School of Bioengineering, College of Engineering and Technology, SRM Institute of Science and Technology, Chennai, Tamil Nadu, India; ^4^Department of Maternity and Child Health Nursing, College of Nursing, Princess Nourah bint Abdulrahman University, Riyadh, Saudi Arabia; ^5^Department of Food Science and Nutrition, CAMS, Sultan Qaboos University, Muscat, Oman; ^6^Ageing and Dementia Research Group, Sultan Qaboos University, Muscat, Oman; ^7^Research and Policy Division, Q3CG Research Institute (QRI), Ypsilanti, MI, United States

**Keywords:** banana, finger millet, fortification, infants, micronutrients, weaning food

## Abstract

Weaning food is a soft, easily digestible type of food other than breast milk for infants aged 6 to 24 months. The present study was conducted to develop cereal-fruit-based complementary foods for infants and evaluate the nutritional quality of such types of foods. Few researchers have focused on formulating weaning foods from locally available, nutritious, and rich ingredients without nutrient loss to reduce malnutrition and infant morbidity rates. In this study, the formulated infant food was prepared from *Musa paradisiaca* (*Nendran* banana) and *Eleusine coracana (ragi*). Formulated weaning food was analyzed using various standard methods, demonstrating that it could provide adequate nutrients to growing infants for their proper growth and development. The shelf life of the weaning food was also studied for a period of 3 months at ambient conditions in two different packaging materials: aluminum and plastic (low-density polyethylene or LDPE), with the aluminum foil pouch exhibiting the best shelf life. This ready-to-serve food, which is formulated and fortified with natural ingredients containing essential macronutrients and micronutrients, could be regarded as highly effective supplementary food for infants. Furthermore, this development has the potential to introduce an affordable weaning product specifically targeted at low socioeconomic groups.

## 1. Introduction

Weaning food refers to a soft and easily digestible source of nutrition aside from breast milk, which is suitable for infants aged 6 to 24 months. It plays a crucial role in providing necessary nourishment for optimal growth and development in infants. An adequate amount of nutrients play a vital role during an infant's maturity phase. The recommended dietary allowance (RDA) establishes the essential nutrient limits that are essential for the growth and development of infants throughout their 1st year of life. As infants grow, they develop the ability to chew and gradually receive a wide variety of complementary foods into their diet, expanding beyond solely consuming liquid nourishment such as breast milk (1). Weaning foods could be comparable to breast milk in terms of ease of digestion. However, breast milk lacks many essential nutrients such as vitamin D, iron, and so on Parvin et al. (2). According to the World Health Organization (WHO), infants should be exclusively breastfed for at least the first 6 months, followed by the introduction of nutritious and complementary foods to achieve healthy, optimal feeding (3). Introducing a combination of cereals, pulses, nuts, fruits, and vegetables during the weaning process can provide infants with a well-rounded diet with adequate calories and nutrients. The RDA also outlines the energy requirements for individuals. The WHO recommends the following RDAs: carbohydrates, 50–95 g; protein, 9.1–11 g; fat, 30–31 g; fiber, 1–4.4 g; vitamin B1, 0.1–0.2 mg; vitamin B2, 0.3–0.4 mg; vitamin B3, 2–4 mg; vitamin B6, 0.1–0.3 mg; vitamin C, 40–50 mg; calcium, 210–270 mg; sodium, 100–200 mg; potassium, 200–700 mg; and iron, 0.27–10 mg (1).

Fruits are an important constituent of the human diet, as they are one of the most important sources of vitamins and minerals. *Musa spp*., comprising banana and plantain, are among the world's leading fruit crops. Worldwide, 125 million tons were produced in 2021, according to the Food and Agriculture Organization (FAO) statistics database (4). Bananas are easily available fruits that are rich in fiber, vitamins, and minerals, which offer great health benefits, especially for babies, making them an excellent supplementary food option. India ranks first among producers in the cultivation of bananas (5). Among the wide varieties of this fruit, Nendran *(Musa paradisiaca)* is considered a supreme variety for its carbohydrate and micronutrient content (6). Nendran banana is typically preferred as a complementary food option since it helps with weight gain and provides the necessary nutrients for babies' growth and development. They are well-known for their texture and aroma and are a good source of potassium, sodium, and calcium (7). Boiled and mashed bananas are an excellent nutrient source for infants, even from 4 months onward. In many countries, mothers choose bananas as the first solid food to introduce to their babies when beginning their solid food intake (8). The soft, mushy texture of bananas helps facilitate easier digestion for babies.

Finger millet (*Eleusine coracana* L.), also known as African millet, is commonly called *ragi* in India. It has excellent nutritional value and functional properties and is superior to other common cereals (9). In other parts of the world, the first food to be introduced to infants is traditionally cereal. Cereals are a major source for the development of weaning food. *Ragi's* mineral content is high when compared to rice and wheat. It is rich in both calcium and iron. No other cereal comes close to *ragi* regarding its calcium content (10). Calcium is an essential micronutrient for children's bone growth and development. *Ragi* can potentially replace calcium pills.

Additionally, it contains B vitamins such as thiamine, riboflavin, and niacin. It is a good source of carbohydrates, protein, and dietary fiber and has a lower natural fat content than other cereals. The fat in *ragi* is unsaturated; hence, it can be used as a substitute for rice and wheat to reduce weight gain (10). All the essential nutrients in *ragi* make it suitable for large-scale production and the manufacturing of different food products. Thus, *ragi* can be used to prepare ready-to-eat or ready-to-cook products to enhance their consumption (11). Therefore, *ragi* is considered one of the most important staple foods for low-income people (11).

Preserving food nutrients with an extended shelf life is challenging for researchers, particularly micronutrients such as vitamins and minerals in bananas and *ragi*. Different drying techniques should be explored to preserve the nutrient content of raw materials. In this study, we used the freeze-drying technique to obtain banana and *ragi* powder to develop weaning food. Freeze drying has been considered the most advanced method of drying, as it helps retain micronutrients without causing any damage to micronutrients and preserves food quality. Therefore, heat-sensitive products can be dried using this method without changing nutritional quality, taste, aroma, flavor, or color. Fortification is the practice of improving the nutritional quality of food products and increasing the content of essential micronutrients to provide high-quality products with minimal risks. Food fortification has been enacted as a public health policy in many countries. Fortification is mainly carried out to ensure that minimum dietary requirements are met. Fortified food reduces the risk of malnutrition in infants, along with breast milk intake. Fortification is an effective way to prevent micronutrient deficiencies. Severe forms of malnutrition change the infant's body structure, physiology, and metabolism.

Consequently, it is essential to add carbohydrates and protein-rich ingredients such as bananas and *ragi* to the weaning food formulation (8). At 6 months of age and beyond, most infants begin to eat supplementary semisolid food. At this stage, fortified weaning food is said to play a major role in their nutrition (12, 13).

In short, weaning food is crucial for the development of infants' physical, brain, and immune systems. Bananas and *ragi* are rich in minerals such as calcium, sodium, potassium, iron, and vitamins such as B1, B2, B3, B6, and C. During the processing of bananas and *ragi*, there is a possibility for a decrease in the number of micronutrients, which is compensated for through fortification in infant food. In addition, bananas and *ragi* lack certain essential vitamins, such as vitamin A, vitamin E, and vitamin B12, that are essential for the growth and development of infants. Therefore, the addition of a premix containing these deficient vitamins per the RDA through fortification was carried out in this research (14).

The aim of this study was to develop nutritionally fortified weaning food from dried banana and *ragi* per RDA and to evaluate the quality of dried weaning food at atmospheric conditions using aluminum and low-density polyethylene (LDPE) pouches as packing materials.

## 2. Materials and methods

### 2.1. Sample preparation and weaning food development

Banana (7th stage), *ragi* flour, skimmed milk powder, whey protein, and dates powder were collected from the local market in Potheri, Chennai, Tamil Nadu, India. The edges of the banana were trimmed and cut into two pieces from the center. It was then steam-cooked at 100°C ± 3°C by indirect steaming for 10 min over medium heat. The bananas were peeled off and slit to remove the seeds from the center. Then, the pieces were transferred into a mixer jar and pulsed once. Afterward, water was added to obtain the desired consistency. The obtained puree was dried using the freeze-drying technique. The steam-cooked banana pulp was pre-frozen by spreading as a 3-mm layer inside a deep freezer at −18°C for 2 days. The pre-frozen sample was placed inside a freeze drier and then automatically dried under vacuum (10 Pa absolute pressure) in the freeze drier (Lyodel,153-06-10, India) at −45°C for 16 h. *Ragi* flour was boiled similarly on medium heat until a porridge-like consistency was obtained. Then, the thick porridge was transferred onto three plates and spread uniformly for freeze-drying per the above protocol. A study conducted by Kabeer et al. (5) indicated that drying can increase the quality and shelf life of bananas. Therefore, following the RDA, both dried powders and other ingredients, such as skimmed milk powder, whey protein, and dates powder, were thoroughly mixed in three different ratios based on their major nutrient content. Subsequently, the most optimal formulation was chosen based on proximate composition and micronutrient analyses.

### 2.2. Proximate analysis

Proximate analysis was performed on the formulated weaning foods. Moisture content was determined by drying the samples at 104°C in a hot air oven until a constant weight was attained (15). Carbohydrate content was determined using the anthrone method (16). Moreover, acid hydrolysis was carried out to break down polysaccharides into simple sugars. The resultant monosaccharides were estimated using a spectrophotometer at a wavelength of 670 nm. Protein content was determined using the Kjeldahl method (17). This method determines the nitrogen content of the sample. The amount of protein was calculated by multiplying nitrogen content with a 6.25 conversion factor. The fat content of the samples was determined using the Soxhlet extraction method (18). Fat extraction was conducted using hexane as the solvent. After 6 h, fat content was measured. The dietary fiber of the samples was determined using acid-alkali hydrolysis (19). Ash content was determined using a muffle furnace. After removing moisture content, samples were kept in the muffle furnace at 500°C for 3 h (20). Each analysis was conducted in triplicate, and values were expressed on a dry-weight basis.

### 2.3. Micronutrient analysis

#### 2.3.1. Determination of minerals

Mineral contents from the formulated weaning food were analyzed with the use of a flame emission atomic absorption spectrophotometer (AAS) at a wavelength of 598 nm. Moreover, 10 g of each formulated sample was placed in a porcelain dish. Then, it was placed inside a muffle furnace at 500°C for 5 h. The samples were transferred into a 250-ml beaker and mixed with 50 ml of deionized water and 15 ml of concentrated nitric acid. The beaker was then heated over a hot plate. Then, the samples were filtered and brought to a final volume of 250 ml by adding deionized water. The standard stock solution of 100 ppm was prepared. The atomic absorption instrument was set up. Then, samples, along with blanks and standards, were read at 589 nm using the AAS. The mineral content was statistically analyzed by developing a calibration curve. The analysis was conducted in triplicate, and values were expressed on a dry-weight basis.

#### 2.3.2. Determination of vitamins

The formulated weaning food contained vitamins such as B1, B2, B3, B6, and C; this vitamin content was analyzed through high-performance liquid chromatography (HPLC). Standards for vitamins B1, B2, B6, and C were prepared by accurately weighing 10–20 g of vitamin powder in 1 ml of deionized water, while for vitamin B3, the standard was prepared by adding 10–20 g of vitamin powder to 0.5 ml of potassium hydrogen carbonate. Working samples were prepared by accurately weighing 0.100 g of dried powder, adding 80 ml of water, and mixing properly. Then, the samples were centrifuged at 4,000 rpm for 25 min. Afterward, the supernatant was collected for vitamin analysis along with the standard. The sample solution was filtered through a 0.25-μm filter. The analysis was conducted using Acclaim PA columns with dimensions of 3.0 × 150 mm and a particle size of 3 μm. The column temperature was maintained at 25°C. The mobile phases were a 25-mm phosphate buffer (pH 3.6) and acetonitrile, with a flow rate of 0.5 mL/min. The vitamin content was calculated based on the methods described in other studies (21), with the aid of retention time and peak area. The analysis was conducted in triplicate, and the results were expressed on a dry-weight basis.

### 2.4. Fortification of weaning food

During the processing of bananas and *ragi*, there may be a high probability of losing some vitamins and minerals. Fortification is the process of replenishing lost micronutrients. A recent study (22) has revealed that fortification has a positive impact on the growth of infants. Weaning food contains vitamins such as vitamins B1, B2, B3, B6, and C and minerals (such as sodium, potassium, calcium, and iron), and any loss in these micronutrients in food can be added externally via fortification. Some vitamins (such as A, E, and B12) are necessary for the growth of infants but are absent in bananas and *ragi*. Thus, they must be added externally as fortifications to meet the RDA. Food-grade vitamins and minerals were obtained from Hexagon Nutrition (Exports) Private Limited in Chennai, Tamil Nadu, India, and used for fortifying the formula.

### 2.5. Physiochemical analysis

Various important physical properties such as water activity, water holding capacity, viscosity, bulk density, and the pH of the selected weaning food were determined. Water activity was measured using a water activity meter (Novasina LabSwift-AW, India) available at the Department of Food and Process Engineering, SRMIST. The water holding capacity was determined by placing 1 g of dried sample into a centrifuge tube containing 10 ml of distilled water with proper mixing. It was then centrifuged at 5,000 rpm for 25 min. The supernatant was discarded, and the residue was weighed with the centrifuge tube. The weight difference provided the value of the water's holding capacity.

Viscosity was determined by mixing 3 g of weaning food with 27 ml of distilled water at 30°C. This slurry was used for determining viscosity. Viscosity was measured in Brook's field viscometer using spindle number 18, rotating at 100 rpm. Bulk density was represented as the ratio of the mass of the sample to the volume of the sample (23). The pH was determined by putting 1 g of the sample in a centrifuge tube containing 10 ml of distilled water and mixing it appropriately. It was then centrifuged at 3,000 rpm for 15 min. The supernatant was collected for pH determination and measured using Systronics digital pH meter 335 (24).

### 2.6. Shelf-life analysis

The shelf life of weaning food was studied for 3 months at 32° ± 3°C by packing the product in two different packaging materials: aluminum and plastic (low-density polyethylene, LDPE). Proximate, micronutrient, physiochemical, and microbial analyses were also part of the shelf-life study. Proximate micronutrient and physiochemical measurements were conducted in accordance with the above procedures. Microbial analysis of the weaning food was performed to assess the bacterial, fungal, and yeast load under laboratory conditions. All media and equipment were sterilized under steam sterilization at 15 psi for 20 min at 121°C in an autoclave. For analysis, 1 g of each sample was weighed and diluted to 1:10 (1 g in 9 ml of water) with distilled water. A serial dilution was prepared and the spread plate technique was performed. Total plate count (TPC) was determined using a nutrient agar (NA) plate, and for yeast and mold, potato dextrose agar (PDA) plates were used. Samples were spread on the agar plates with the help of glass rods (25, 26). The plates were then incubated at 37°C for 24 to 72 h.

### 2.7. Statistical analyses

The data were analyzed statistically using a one-way ANOVA. All the analysis was conducted in triplicate, and the data were expressed as mean ± SD, and significance was accepted at a *p-*value of < 0.05 per Duncan's multiple range test.

## 3. Results

### 3.1. Development of weaning food

For product development, three different formulations were created from the freeze-dried banana and *ragi* powders (Table 1). Formulations were created based on the RDA requirements. Besides bananas and *ragi*, raw materials remained constant in these three formulations. The best formulation was selected based on the proximate and micronutrient analyses. FB-Formulation 2 fulfilled the necessary RDA criteria.

**Table 1:** Different formulations for preparing weaning foods.

**Ingredients**	**Formulation-I (g/100 g)**	**Formulation-II (g/100 g)**	**Formulation-III (g/100 g)**
Banana powder	45	40	50
*Ragi* powder	35	40	30
Skimmed milk powder	10	10	10
Whey protein	5	5	5
Dates powder	5	5	5

### 3.2. Proximate analysis of weaning food

**Figure 1:**
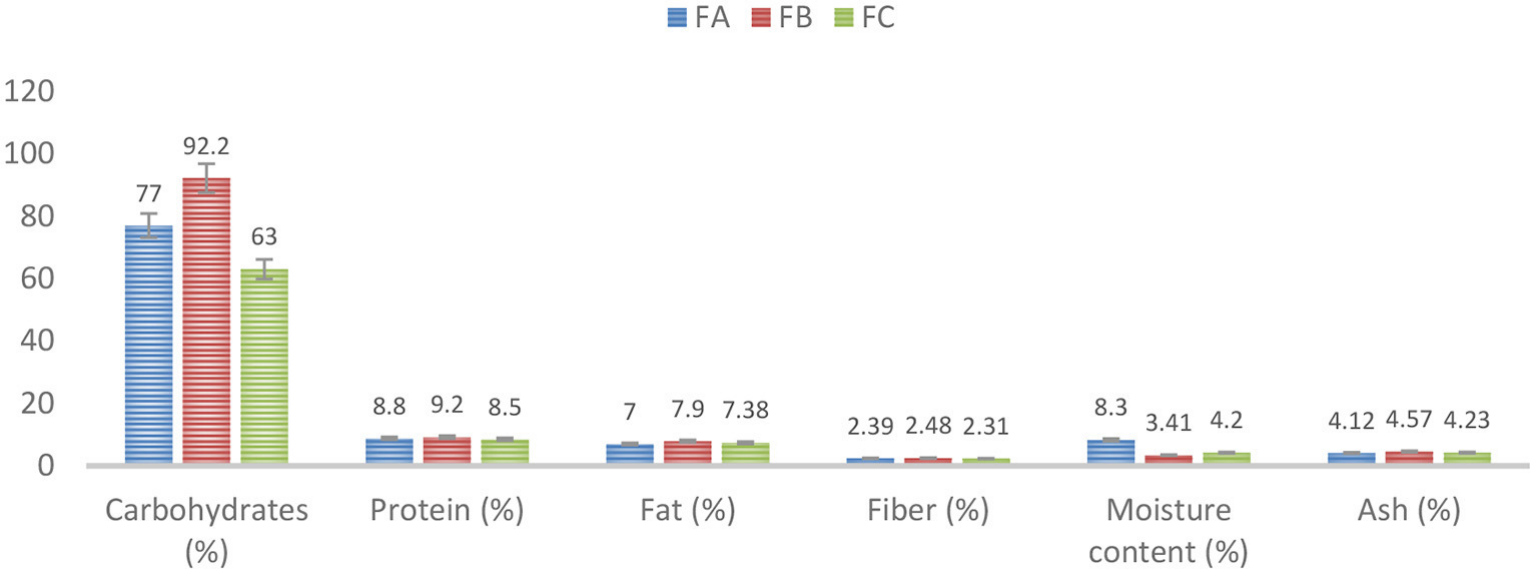
Proximate analysis for different formulations, FA-Formulation 1, FB-Formulation 2, and FC- Formulation.

The results of proximate analysis for different formulations are described graphically in Figure 1. The results showed that the second formulation was the best, as the values of the proximate components of FB-Formulation 2 were very close to those of the RDA. The RDA for the carbohydrate content for infants is between 50 and 95 g/100 g. The carbohydrate content of the formulated weaning food was determined to be 92 g/100 g. The protein content of the formulated weaning food was found to meet the RDA. The RDA for the protein content for infants was estimated to be 9.20 g/100 g. Ash content corresponded to its mineral content. The formulated weaning food had a high amount of ash content (4.57 g/100 g). The data were analyzed statistically using one-way ANOVA via Minitab. All the analyses were conducted in triplicate, and the data were expressed as mean ± SD, and significance was accepted at a *p-*value of < 0.05 per Duncan's multiple tests.

### 3.3. Micronutrient analysis of weaning food

**Figure 2:**
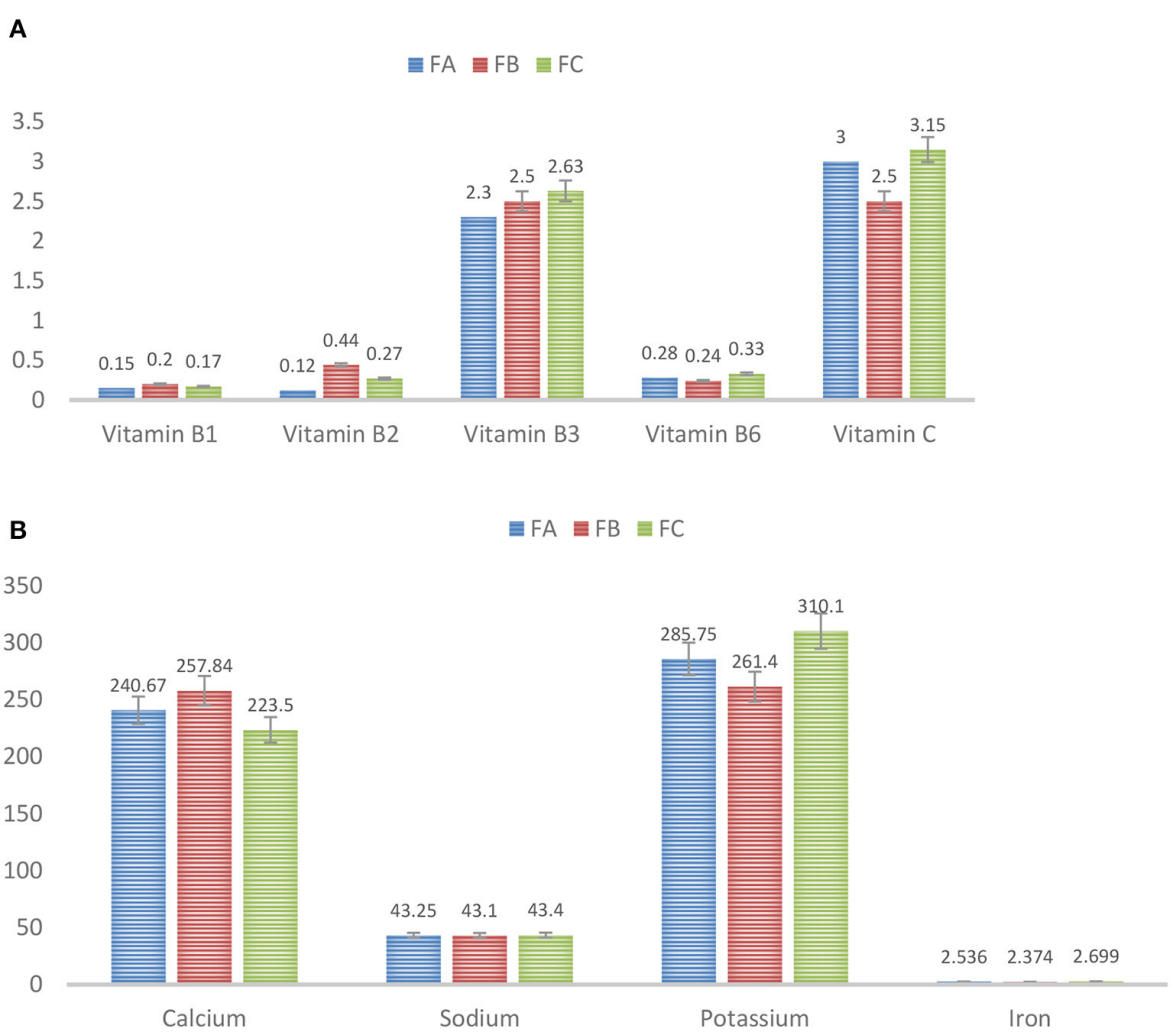
**(A)** Vitamin analysis for different formulations FA-Formulation 1, FB-Formulation 2, and FC- Formulation 3. **(B)** Mineral analysis for different formulations FA-Formulation 1, FB-Formulation 2, and FC- Formulation 3.

Figures 2A, B describes the vitamin and mineral analyses for different formulations of weaning foods. The results indicated that all other micronutrients met the infant's RDA requirements except sodium and vitamin C contents. The results also showed that FB-Formulation 2 was best for providing a weaning infant with adequate micronutrients based on the infant's RDA. The data were analyzed statistically using a one-way ANOVA via Minitab. All the analyses were conducted in triplicate, and the data were as expressed as mean ± SD, and significance was accepted at a *p-*value of < 0.05 per Duncan's multiple tests.

The RDA of sodium content for weaning food was within the range of 1–2 mg/g. However, the sodium content of the formulated weaning food was 431 μg/g. The RDA of vitamin C content was within the range of 40–50 mg/100 g. However, vitamin C content was 250 μg/g in the formulated freeze-dried weaning foods. The possible reason for these trivial values is that the drying banana procedure to obtain a powder reduces the content of both sodium and vitamin C. Therefore, fortification was carried out to increase the nutrient content of the formulated weaning food.

The results indicated that all the other micronutrients met the infant's required RDA except sodium and vitamin C content. The results also confirmed that FB-Formulation 2 was best for developing weaning foods with an adequate number of micronutrients.

### 3.4. Fortification of weaning food

FB-Formulation 2 was selected for the development of fortified weaning foods based on the results of proximate and micronutrient analysis. This formulation contained an equal amount of *ragi*, banana powder, and other ingredients, as mentioned above (Table 1). Owing to certain nutrient insufficiencies, fortification with a premix containing these deficient vitamins as per the RDA was employed.

### 3.5. Shelf-life analysis

**Figure 3:**
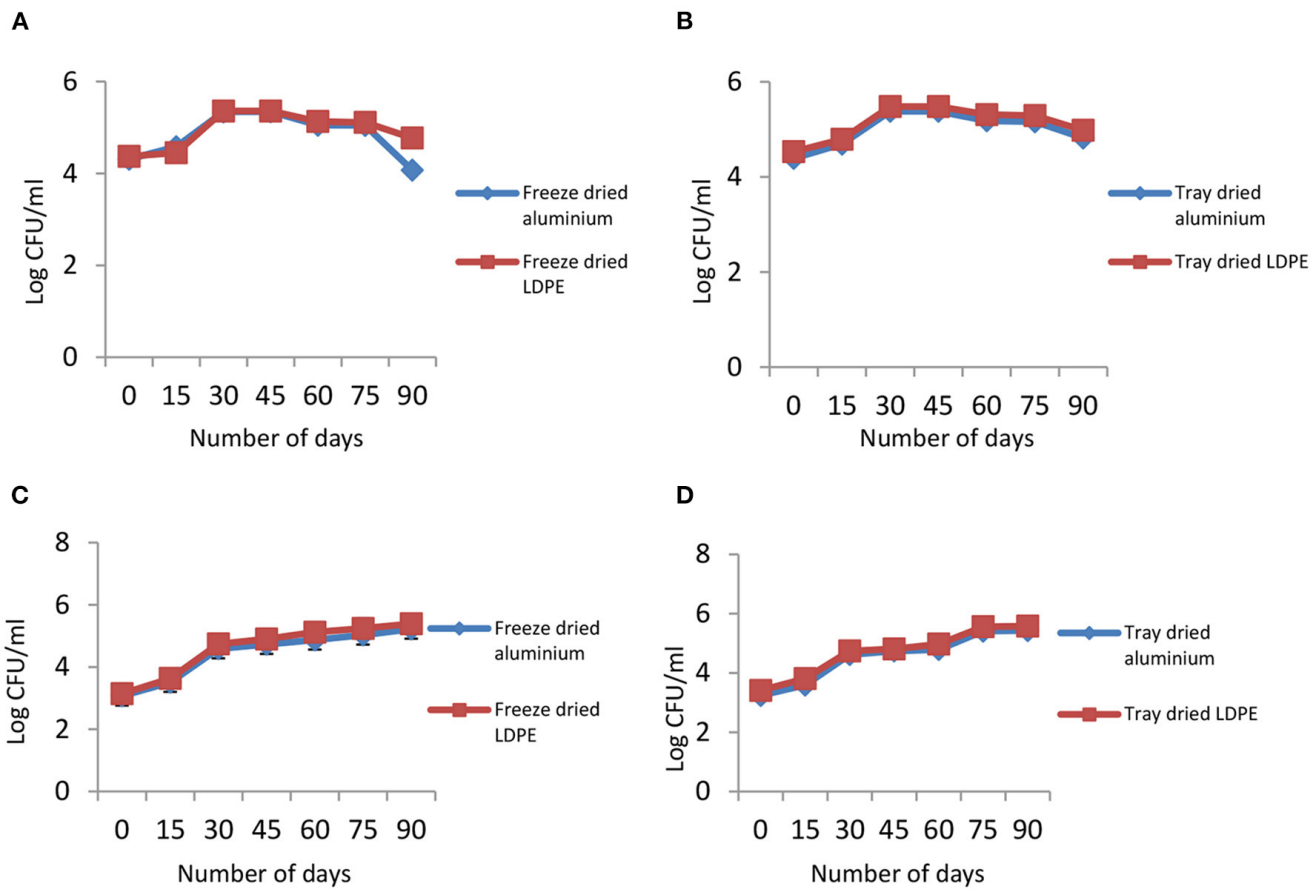
Changes in microbial load during the shelf-life of fortified weaning foods. **(A)** Bacterial growth of freeze-dried weaning. **(B)** Bacterial growth of tray-dried weaning food during shelf-life. **(C)** Fungal growth of freeze-dried food. **(D)** Fungal growth of tray-dried weaning food during shelf-life.

The shelf life of weaning food was studied for 3 months by packing the product in two different packaging materials: aluminum and LDPE. Changes in the functional properties and micronutrient content during the shelf-life study are depicted in Tables 2, 3. The results indicate that changes are detected in carbohydrates, fat, moisture content, water activity, viscosity, and pH values of weaning food packed in different packaging materials. The data were analyzed statistically by one-way ANOVA using Minitab. The significant difference increases when the storage life increases. Statistical analysis revealed that products packed in plastic packaging show a significantly higher increase when compared to products packaged in aluminum. Microbial growth during storage is presented graphically and expressed as log CFU/ml. Figure 3 shows the bacterial and fungal growth of weaning food during its shelf life. The microbial load appeared to be under control upon extended storage and can be considered safe for infants' consumption.

**Table 2:** Changes in the functional properties of freeze-dried weaning food during its shelf life.

**Functional properties**	**Day 0**	**Day 15**		**Day 30**		**Day 45**		**Day 60**		**Day 75**		**Day 90**	
		**Al**	**LDPE**	**Al**	**LDPE**	**Al**	**LDPE**	**Al**	**LDPE**	**Al**	**LDPE**	**Al**	**LDPE**
Carbohydrates (%)	91 ± 0.23^a^	91 ± 0.04^a^	91 ± 0.02^a^	90 ± 0.02^a^	90 ± 0.05^b^	90. ± 0.02^b^	89 ± 0.01^b^	90 ± 0.01^b^	89. ± 0.04^b^	90 ± 0.01^c^	88 ± 0.04^c^	89 ± 0.01^c^	88 ± 0.01^c^
Fat	7.9 ± 0^a^	6.8 ± 0.46^b^	6.8 ± 0.07^b^	6.6 ± 0.02^c^	6.5 ± 0.02^c^	6.3 ± 0.03^c^	6.3 ± 0.01^c^	6.1 ± 0.05^c^	6.1 ± 0.01^c^	6.1 ± 0.04^c^	5.9 ± 0.01^c^	6.0 ± 0.05^c^	5.9 ± 0.05^c^
Fiber (%)	2.4 ± 004^a^	2.4 ± 0^a^	2.4 ± 0.1^a^	2.4 ± 0.07^a^	2.3 ± 0.08^b^	2.3 ± 0.08^b^	2.3 ± 0.03^b^	2.3 ± 0.1^b^	2.3 ± 0.2^b^	2.3 ± 0.4^b^	2.3 ± 0.1^b^	2.3 ± 0.1^b^	2.2 ± 0.1^b^
Ash content (%)	4.5 ± 0.04^a^	4.4 ± 0.35^a^	4.4 ± 0.21^a^	4.4 ± 0.08^b^	4.3 ± 0.1^b^	4.3 ± 0.2^b^	4.2 ± 0.07^c^	4.3 ± 0.47	4.2 ± 002^c^	4.2 ± 0.01^c^	4.1 ± 0.041^c^	4.2 ± 0.02^c^	4.16 ± 0.03^c^
Moisture content (%)	3.4 ± 0.21^a^	3.4 ± 0.012^a^	3.4 ± 0.01^a^	3.9 ± 0.05^b^	4.21 ± 0.01^b^	4.53 ± 0.04^b^	4.98 ± 0.07^b^	4.78 ± 004^b^	5.34 ± 0.1^c^	4.8 ± 0.02^b^	5.38 ± 0.12^b^	4.82 ± 0.5^c^	5.4 ± 0.31^c^
Bulk density (g/cm3)	0.56 ± 0.4^a^	0.404 ± 24^a^	0.42 ± 23^a^	0.40 ± 0.24^a^	0.42 ± 0.4^a^	0.40 ± 0.21^a^	0.424 ± 21^a^	0.404 ± 13^a^	0.42 ± 0.1^a^	0.35 ± 0.1^b^	0.424 ± 24^a^	0.35 ± 0.4^b^	0.424 ± 0.5^a^
Water holding capacity	9.62 ± 0.36^a^	9.62 ± 37^a^	9.29 ± 14^a^	9.54 ± 0.6a	9.21 ± 14^a^	9.52 ± 0.45^a^	9.14 ± 24^a^	9.47 ± 32^a^	9.12 ± 0.1^a^	9.39 ± 23^a^	8.25 ± 13^b^	9.39 ± 0.8^a^	8.25 ± 0.4^b^
Water activity (aw)	0.42 ± 0.35^a^	0.42 ± 0.5^a^	0.42 ± 0.28^a^	0.48 ± 0.9^a^	0.494 ± 32^a^	0.45 ± 0.9^a^	0.478 ± 31^a^	0.50 ± 0.1^a^	0.52 ± 0.1^b^	0.504 ± 17^b^	0.543 ± 10^b^	0.50 ± 0.6^a^	0.51 ± 0.2^a^
Viscosity (cP)	1.05 ± 0.4^a^	1.5 ± 0.7^b^	1.56 ± 0.6^b^	1.52 ± 0.4^b^	1.62 ± 0.4^b^	1.86 ± 0.4^b^	2.31 ± 2.1^c^	2.38 ± 0.4^c^	2.6 ± 0.31^c^	2.52 ± 0.9^c^	2.86 ± 0.1^c^	2.13 ± 0.1^c^	2.54 ± 0.8^c^
pH	6.19 ± 0.2^a^	6.19 ± 0.4^a^	6.29 ± 0.4^a^	6.34 ± 0.2^a^	6.4 ± 1.8^a^	6.36 ± 0.4^a^	6.46 ± 1.3^b^	6.32 ± 0.6^a^	6.46 ± 2.3^b^	6.36 ± 1.6^a^	6.48 ± 0.7^b^	6.36 ± 0.3^a^	6.49 ± 0.4^b^

Each data point represents the mean ± standard deviation of triplicate analysis. The multiple comparisons were determined using Duncan at a *p*-value of < 0.05 columns with significantly different letters (*p* < 0.05).

**Table 3:** Changes in the micronutrient content of freeze-dried weaning food during its shelf life.

**Micronutrients (mg/100 g)**	**Day 0**	**Day 15**		**Day 30**		**Day 45**		**Day 60**		**Day 75**		**Day 90**	
		**Al**	**LDPE**	**Al**	**LDPE**	**Al**	**LDPE**	**Al**	**LDPE**	**Al**	**LDPE**	**Al**	**LDPE**
Vitamin B1	0.2 ± 0.04^a^	0.2 ± 0.01^a^	0.2 ± 0.1^a^	0.2 ± 0.2^a^	0.2 ± 0.1^b^	0.2 ± 0.2^b^	0.1 ± 0.1^c^	0.1 ± 0.1^c^	0.1 ± 0.4^c^	0.1 ± 0.1^c^	0.1 ± 0.5^c^	0.1 ± 0.2^c^	0.1 ± 0.1^c^
Vitamin B2	0.5 ± 0.03a	0.5 ± 0.03^a^	0.48 ± 0.3^b^	0.517 ± 0.3^a^	0.41 ± 0.3^b^	0.4 ± 0.01^b^	0.3 ± 0.2^c^	0.4 ± 0.2^b^	0.3 ± 0.1^c^	0.4 ± 0.1^b^	0.3 ± 0.7^c^	0.41 ± 0.3^b^	0.31 ± 0.6^c^
Vitamin B3	2.5 ± 0.12^a^	2.45 ± 0^a^	2.43 ± 0.5^a^	2.42 ± 0.1^a^	2.37 ± 0^b^	2.35 ± 0.1^a^	2.3 ± 0.04^b^	2.34 ± 2^a^	2.26 ± 3^b^	2.31 ± 2^b^	2.22 ± 7^b^	2.29 ± 1^b^	2.16 ± 0.1^c^
Vitamin B6	0.24 ± 0.04^a^	0.23 ± 0.2^a^	0.19 ± 0.3^b^	0.21 ± 0.62^a^	0.17 ± 0.1^b^	0.19 ± 0.1^b^	0.15 ± 0.1^c^	0.186 ± 0.2^b^	0.134 ± 0.4^c^	0.18 ± 0.1b	0.13 ± 0.4^c^	0.16 ± 0.1^c^	0.13 ± 0.7^c^
Vitamin C	43.5 ± 0.05^a^	43.2 ± 0.1^a^	43.1 ± 0.1^a^	43.1 ± 0.1^a^	43.1 ± 0.1^a^	43.1 ± 0.2^a^	43.0 ± 0.1^a^	43.0 ± 0.1^a^	42.5 ± 0.1^b^	42.9 ± 0.6^b^	42.1 ± 0.3^b^	42.8 ± 0.1^c^	42.0 ± 0.2^c^
Vitamin A	450 ± 0.1^a^	449.9 ± 0^a^	449.7 ± 0.3^a^	449.7 ± 0.4^a^	449.2 ± 0.7^a^	448.6 ± 0.3^b^	448.3 ± 0.2^b^	447.6 ± 0.4^c^	447.3 ± 0.7^c^	446.0 ± 0.1^c^	445.9 ± 0.7^c^	445.9 ± 0.3^c^	445.6 ± 0.7^c^
Vitamin B12	0.45 ± 0.1^a^	0.42 ± 0.1^a^	0.421 ± 0.4^a^	0.413 ± 0.1^b^	0.419 ± 0.4^b^	0.413 ± 0.1^b^	0.406 ± 0.1b	0.412 ± 0.1^c^	0.404 ± 0.4^c^	0.413 ± 0.7^c^	0.404 ± 0.1^c^	0.413 ± 0.4^c^	0.401 ± 0.1^c^
Vitamin E	4.5 ± 0.1^a^	4.48 ± 0.1^a^	4.45 ± 0.3^a^	4.45 ± 0.1^a^	4.45 ± 0.1^a^	4.42 ± 0.1^b^	4.40 ± 0.1^b^	4.40 ± 01^b^	4.40 ± 0.5^b^	4.395 ± 0.1^c^	4.33 ± 0.01^c^	4.37 ± 0.05^c^	4.31 ± 0.04^c^
Calcium	257.84 ± 0.01^a^	257.4 ± 0.1^a^	257.2 ± 0.1^a^	257.1 ± 40.3^a^	257 ± 0.1^a^	257 ± 0.4^a^	256.9 ± 0.1^b^	257 ± 0.2^a^	256.9 ± 0.5^b^	256.9 ± 0.9^b^	256.8 ± 0.1^b^	256.8 ± 0.2^c^	256.7 ± 0.1^c^
Sodium	143.2 ± 0.01^a^	142.8 ± 0^a^	142.6 ± 0.9^a^	142.5 ± 0.1^a^	141.9 ± 0.5^b^	142.06 ± 0.8^a^	141.09 ± 0.1^b^	141.2 ± 0.1^b^	140.2 ± 0.4^c^	140.3 ± 0.1^b^	139.5 ± 0.7^c^	140.3 ± 0.1^b^	139.4 ± 0.6^c^
Potassium	261.4 ± 0.01^a^	261.3 ± 0.1^a^	261.1 ± 0.1^a^	261.24 ± 0.23^a^	261.1 ± 0.7^a^	261.2 ± 0.4^b^	261.04 ± 0.2^b^	261.1 ± 0.1^b^	261 ± 0.21^b^	261 ± 0.12^b^	260.9 ± 0.1^b^	260.9 ± 0.1^b^	260.8 ± 0.2^b^
Iron	2.31 ± 0.04a	2.3 ± 0.1^a^	2.28 ± 0.04^a^	2.29 ± 0.1^b^	2.21 ± 0.1^a^	2.27 ± 0.3^b^	2.1 ± 0.5^b^	2.2 ± 2^b^	2.15 ± 24^c^	2.2 ± 0.14^c^	2.11 ± 1^c^	2.18 ± 123^c^	2.1 ± 0.24^c^

Each data point represents the mean ± standard deviation of triplicate analyses. The multiple comparisons were determined using Duncan at a *p-*value of < 0.05 columns, with the different letters being significantly different (*p* < 0.05).

### 3.6. Figures and tables

The series of figures and tables presented below describe our research findings and are referenced in the text in the Results section.

## 4. Discussion

An infant's firs1stt year is crucial for its growth and development. Typically, in the first 6 months, growth and weight gain are pronounced, while in the second half of the first year, they are not as rapid. Infants experience a wide range of developmental milestones in interacting, learning, speaking, behaving, and movement (1–3). Adequate nutrition is vital for brain development, especially during pregnancy and infancy. The introduction of complementary feeding, i.e., semisolid weaning food around 6 months of age and breast milk feeding or infant formula, is encouraged and highly recommended. In low-income countries (LICs) or lower-middle-income countries (LMICs), as well as low socioeconomic groups, good-quality weaning foods or practices are a tremendous challenge. This presents a real dilemma for mothers regarding infant feeding and maintaining a suitable nutritional status. Locally sourced ingredients potentially mitigate nutritional risks (4–8).

For example, India has become the world's most populous country. The 2021 gross national income (GNI) per capita is between $1,086 and $4,255. After mangoes, banana variety production is India's most important fruit crop. On average, the output is ~29 million tons per year, mostly serving the domestic market (FAO website) (4, 5). Considering its market availability and nutritional composition, bananas offer an opportunity to develop affordable, appropriately formulated, fortified weaning food for infants for local market consumption.

The process of formulating weaning food involves two crucial components: product development, quality control, and regulatory approval. First, it entails determining the nutrient content of the formulation, which includes analyzing the proximate composition, and micronutrient constituents and implementing fortification. Second, it involves assessing and ensuring the shelf life of the developed product.

Ingredients for the weaning food formulation were sourced locally. Three different formulations were explored: FA-Formulation 1, FB-Formulation 2, and FC-Formulation 3. Sample preparation and mix ratios of constituents are provided in detail above (Table 1) (5). The best formulation was selected based on the proximate composition (a measure of moisture, ash, lipid, protein, and carbohydrate contents) and micronutrient analyses (a measure of vitamins and minerals). Based on the analytical findings, Formulation 2 was deemed best and was selected for further studies [proximate analysis, (Figure 1) and vitamins & minerals analyses, (Figure 2)] utilizing two different packaging materials, aluminum and LDPE (Tables 2, 3), prepared under freeze-dried and tray-dried conditions.

In the following paragraphs, the discussion will focus on two processes involved in the development of weaning foods. In the future, we will ascertain the optimal formulation/fortification and appraise the quality content investigation part before discussing experimental shelf-life interpretation.

### 4.1. Formulation and content assessment

The freeze-dried FB-Formulation 2 weaning food carbohydrate content was 92 g/100 g, while the tray-dried one was 57 g/100 g (data not shown), which is within the value limits of RDA criteria. The RDA recommendation for carbohydrates for infants is 50–95 g/100 g. The protein content for both methods was found to be nearly identical and meet the RDA requirement. Even though freeze-dried weaning food had a slightly higher protein content (data not shown), the ash content, which reflects the mineral content of the formulation, was somewhat higher in the freeze-dried weaning food compared to the tray-dried weaning food. However, it is important to consider that a high fiber intake may not be advantageous for infants, as their nutritional needs differ in terms of fiber requirements (2). The formulated weaning food had a low fiber content, which, although low, met the suggested RDA value. Comparatively, the weaning formulation contained a lower amount of fat compared to the RDA guidance. However, a food sample with a high fat content increases the risk of spoilage by more than one for a food sample with a lower fat content (27). Hence, a low-fat content reduces the possibility of spoilage and increases shelf life. Moisture content is also an important factor in preserving food products for a longer period (28). FB-Formulation 2 had the least moisture content.

Micronutrient contents met the standard values provided by the RDA except for sodium and vitamin C. The recommended sodium content range is 100–200 mg/100 g. In the formulated freeze- and tray-dried weaning food, sodium content was 43.1 and 42.8 mg/100 g (not shown), respectively. The recommended vitamin C content range is 40–50 mg/100 g. Vitamin C content was 2.5 and 2.1 mg/100 g (not shown) in the formulated freeze- and tray-dried weaning foods. One possible explanation is that freeze-dried bananas reduces the content of both sodium and vitamin C. Generally, freeze-dried powder retains higher amounts of sodium and vitamin C than tray-dried powder. Therefore, fortification was implemented to increase the content of sodium and vitamin C in formulated weaning foods. Besides, the formulation was enriched with vitamins A, E, and B12, as they are absent in bananas and *ragi*.

### 4.2. Shelf-life evaluation

Shelf-life experiments on weaning FB-Formulation 2 were conducted on two different packaging materials, aluminum and LDPE, which were prepared either under freeze-dried or tray-dried protocols. Only freeze-dried results are displayed.

The formulated weaning food initially showed the highest bulk density before declining over time. Usually, the higher the bulk density, the lower the moisture content (29). This is a direct indicator of the best storage life for the powder. These results were corroborated by other reports (30). Water holding capacity corresponded with the ability of the powder to absorb water and attain a desired consistency. Formulated weaning food had high water holding capacity, and no significant change was noticed during the storage period, which has also been confirmed previously by other reports (31).

For the freeze-dried concoction, there were no significant changes in the macronutrient (carbohydrate, protein, fat, and fiber) and mineral (ash) contents except for the moisture content value. We observed that the moisture content increased from 4.5% to 6.81%. The products packed in LDPE packaging material showed a significant change in the proximate content during shelf-life experiments when compared to aluminum packaging material. It increased from 3.4% to 5.4%, while aluminum-packed products increased from 3.4% to 4.82%. This can be explained by the fact that plastic is more susceptible to light, oxygen, and moisture than aluminum packaging and that high moisture content encourages the growth of microorganisms (25).

In most cases, storage temperature reduces the protein content. However, in this study, the weaning food was stored at room temperature, causing fewer changes in the gross protein content. Fat content typically undergoes oxidation during the storage period, leading to rancidity (32). Even so, the presence of vitamin E (more than 50%) reduces the rancidity caused by fat oxidation. It is possible that vitamin E, an antioxidant, reduces fat oxidation, thereby diminishing off-flavor development (27). Carbohydrates are relatively more stable under storage conditions compared to vitamins. There was no significant loss in the nutritional value of carbohydrates during the storage period. The same effect was also noted by Abdullah et al. (28) in dried foods. Distinctively, water-soluble vitamins were more sensitive than fat-soluble vitamins during the storage period (30).

Nevertheless, vitamin B loss during the storage period appeared to be insignificant. However, decreases in the contents of vitamins B1, B2, and C were noticed. These vitamins are highly unstable to light, moisture, and oxygen; subsequently, products packed in LDPE had more vitamin content depletion than those packed with aluminum. There was no detectable alteration in vitamin A content. With regards to mineral content, there was no evident quantifiable variation in this study, which is consistent with other investigations (30).

Reduced viscosity is one of the quality attributes for weaning food preparation (33). Viscosity represents the pasting property of food, which shows no changes in the product's texture. Furthermore, it emphasizes high caloric density per unit volume of food. The formulated freeze-dried weaning food had a low viscosity that slightly rose over storage time. This change was not statistically significant. The preliminary pH of the formulated weaning food was 6.19. It remained acidic during the entire storage period, rising negligibly and never attaining alkalinity. Less acidic foods are easier to digest by infants as their digestive systems are immature (30, 33).

Water activity measures the amount of water present in the product. The higher the water activity, the greater the chance for the growth of microorganisms. In the beginning, the water activity was very low; then, it gradually surged due to adjustments in moisture content (33, 34). In this present study, the overall bacterial count of weaning food was observed to be satisfactory per Food Safety and Standard Authority of India regulations (35). Initially, the count was low, but gradually, it increased, became constant, and then declined. The fungus counts increased during the storage period due to the high moisture content. Fungal growth was more prevalent in the product packed in LDPE (36). A study conducted by Gull et al. (10) discussed the development of weaning food from *ragi* flour, green gram flour, and rice flour and established that *ragi* could be considered a good and cheap source of protein, fat, fiber, calcium, and iron for young children. In their study, they conducted microbial analysis and calculated the standard plate count, which is negligible.

## 5. Conclusions

Malnutrition is a serious issue and is prevalent worldwide, especially infant malnutrition. The current study demonstrates that both banana and *ragi* freeze-dried weaning formulations can provide the necessary nutrients for an infant's growth and development. The nutritional value of bananas is remarkable due to their high caloric, vitamin, and mineral contents. *Ragi* powder can be used to make porridge for infants and offers several advantages. To boost the nutritional value of the weaning food, adjustments must be made to its formulation, handling, and storage. Overall, the developed weaning food meets RDA values for all necessary nutrients except sodium and vitamin C. The formulated weaning food was fortified with a food-grade premix to improve product quality, and its RDA compliance was confirmed. The selected formulated freeze-dried weaning food was determined to be nutrient-rich and microbially safe for infants. This ready-to-serve weaning food could be considered an effective supplementary food for infants as it consists of natural ingredients and is enriched with micronutrients. These formulation findings may yield a reasonable solution for low-socioeconomic groups.

## Data availability statement

The original contributions presented in the study are included in the article/supplementary material, further inquiries can be directed to the corresponding authors.

## Author contributions

SK carried out the experiments and wrote the manuscript draft. NG designed, executed, and supervised the study. MQ conducted the content evaluation and critical scientific and technical editing of the manuscript. PR, HA, ME, MQ, and NG intellectually contributed and edited the final manuscript. All authors contributed to the article and approved the submitted version.
